# Body dissatisfaction and dieting among Finnish adolescents: a 20-year population-based time-trend study

**DOI:** 10.1007/s00787-023-02327-0

**Published:** 2024-01-02

**Authors:** Kaisa Mishina, Kim Kronström, Emmi Heinonen, Andre Sourander

**Affiliations:** 1 Department of Child Psychiatry, Research Centre for Child Psychiatry, University of Turku, Lemminkäisenkatu 3, 20014 Turku, Finland; 2 INVEST Research Flagship, University of Turku, Turku, Finland; 3 Department of Adolescent Psychiatry, University of Turku, Turku, Finland; 4 Department of Child Psychiatry, Turku University Hospital, Turku, Finland

**Keywords:** Adolescent, Body dissatisfaction, Body image, Dieting, Eating disorder, Time-trend

## Abstract

Body image has an important role in the health and development of adolescents. Body dissatisfaction and dieting can become chronic and continue into adulthood. There is a lack of recent, comprehensive studies on ongoing trends on body dissatisfaction. This study assessed time-trend changes in self-reported body dissatisfaction and dieting among Finnish adolescents at four assessment points. Representative samples of adolescents (*N* = 6660) aged 13–16 years participated in school-based, cross-sectional studies in 1998 (*N* = 1458), 2008 (*N* = 2044), 2014 (*N* = 1809), and 2018 (*N* = 1349), respectively. The studies were similar in design, methodology, and geographical recruitment areas. Body dissatisfaction and dieting were assessed with a questionnaire including items derived from the DSM-IV criteria for anorexia and bulimia nervosa. From 1998 to 2018, dieting and fears related to gaining weight decreased among females. Body dissatisfaction reduced among females, and their wishes to become thinner became less prevalent. Consuming large amounts of food at one time consistently decreased among females and males and there were no changes in the rates of willful vomiting. The number of females in the 90th percentile with the most severe symptoms decreased. The results indicate that body dissatisfaction and disturbed dieting improved among females during the 20-year study. Despite these positive developments, the overall level of symptoms among females remained substantial, indicating that females have much higher levels of body dissatisfaction and dieting than males.

**Supplementary Information:**

The online version contains supplementary material available at 10.1007/s00787-023-02327-0.

## Introduction

Body dissatisfaction, defined as having negative thoughts and feelings about one’s own body, has an important role in the health and development of adolescents [1, 2]. Adolescence is a vulnerable time, and satisfaction with one’s own body often becomes a significant component of a person’s identity during this period [1, 3]. Body image and eating behaviors can be shaped by current culture and media influences, athletics, and personal relationships [4]. Modern Western society has typically promoted certain body shapes: females are ideally slim, and males are slender and moderately muscular [2].

Body dissatisfaction is common and increases linearly when children reach adolescence [5], just when outside pressures and peer influences grow [6]. As the use of social media has increased exponentially during the last years and is expected to continue to grow substantially [7], adolescents will most likely continue to face this kind of pressure. Currently, over 90% of adolescents use social media daily [8]. As physical appearance and idealized images are central in social media [9], they can further foster body dissatisfaction [10, 11]. One central reason for this is that idealized images can activate a social comparison of one’s own appearance to that of others, including unrealistic body appearances [11]. Viewing and following social media influencers and celebrities is also common, which can also increase social comparison and intentions to change one’s own appearance [12]. In addition, the internet is a continually evolving and ubiquitous source of information [13] with fitness and nutrition as the top searches for health information among adolescents [8].

Body dissatisfaction is present among adolescents, regardless of gender. These concerns include for example muscularity-oriented disordered eating behavior [14], wishes to be thinner and attempts to lose weight [15]. Further, problematic eating attitudes and behavior are common [16, 17] and can be precursors for future eating disorders [18]. In fact, body dissatisfaction may continue into adulthood, as several personal factors, such as weight concerns and weight importance during adolescence often predict disordered eating in young adulthood [19]. Thus, to increase early identification and prevention as well as to develop low-threshold treatment services, body dissatisfaction and dieting among adolescents should be assessed before the symptoms exceed diagnostic levels.

Secular trends in self-reported body dissatisfaction and dieting among adolescents in the general population have been reported in some time-trend studies between the 1990s and the 2010s with partly contradictory findings. These studies have reported improved or stable rates in body image [17, 20, 21] and increases in body satisfaction [19]. Studies have also reported relatively stable rates in eating disorder symptoms [22] and in eating attitudes and behaviors among adolescents [17]. Other studies have indicated increases in dieting in males and females [23, 24] and that the number of adolescents concerned about their weight has increased [25]. Thus, it seems that there is no clear trend for body dissatisfaction or weight concerns. However, this may be partly explained by studies having different years of assessment, or having studies with rather short interval between cross-sectional studies. The heterogeneity of the current knowledge further supports the need to have recent results about the topic.

Body dissatisfaction and disturbed eating, such as problematic dieting can have a major effect on adolescent development. Rapid behavioral and social changes such as the growing use of social media may have an impact on body dissatisfaction among this age group, as its usage is a plausible risk factor for the development of eating disorders [26]. Therefore, comprehensive studies on these trends are important. The aim of this study was to assess time-trends in self-reported body dissatisfaction and dieting from 1998 to 2018 in a nationally representative sample of Finnish adolescents aged 13–17 years old. The research question asked what kind of time-trend changes could be observed regarding body dissatisfaction and dieting among adolescents. Compared to most previous studies, this study benefits from multiple assessment points that span over a long period of time. The results are based on four similar comparative cross-sectional studies, with identical designs, methods and assessment procedures, and that were carried out in geographically well-defined school districts. The geographical areas were from northern and southern Finland, which favors the generalizability of the findings. Based on the results of a previous study of Finnish adolescents [22], we expected that body dissatisfaction and dieting among the adolescents would remain quite steady over time.

## Methods

### Subjects and procedure

The cross-sectional survey was conducted in two Finnish cities, Rovaniemi and Salo, in 1998, 2008, 2014, and 2018. All adolescents in the 7th and 9th grades (age range 13–17, mean age = 14.4, SD = 1.1) of every secondary school (lower secondary school, middle school) in Rovaniemi and Salo were invited to participate in the study. According to Finnish statistics [22], the sex distribution, ethnic backgrounds and family compositions of the communities were comparable to the national demographics for this age group in Finland.

Rovaniemi and Salo represent the northern and southern regions of Finland, respectively. Both cities consist of large geographical areas and diverse communities including urban and rural areas, which is typical of Finnish cities. Rovaniemi has a total population over 60,000. Salo has a population of over 50,000 [27]. Due to the reformation of the municipality system in Finland, the former municipalities of Salo and Rovaniemi have merged with other municipalities. These new municipalities were not included in this study; only the areas that participated in the four studies were included.

**Fig. 1:**
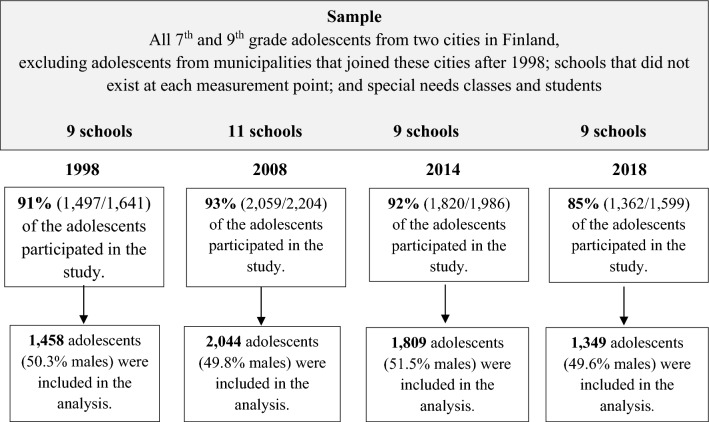
Flow chart

The questionnaire was distributed to adolescents in their classrooms during a normal school lesson. The adolescents filled out the questionnaire anonymously and returned it to the teacher in a sealed envelope. The teacher then sealed the returned envelopes in a bigger envelope in the presence of the students and returned it to the research group. All adolescents who were present at school on the day of the survey were asked to participate. Teachers were also given instructions to ask the students who were absent that day to complete the questionnaire later. Despite this, the majority of the non-respondents were those who were absent from school on the actual survey day. In 1998, 144/1,641 adolescents did not participate; 145/2,204 in 2008; 166/1,986 in 2014; and 237/1,599 in 2018. This resulted in a total of 6,738 returned questionnaires. Of those, 78 were excluded due to incomplete or inappropriate answering (e.g., if a questionnaire consisted only or mostly of swear words or inappropriate drawings): 39 in 1998; 15 in 2008; 11 in 2014; and 13 in 2018. Figure 1 presents the flow for each year.

The ethical standards from the 1964 Declaration of Helsinki and its later amendments were followed. The study was approved by the Ethical Committee at the Hospital District of Turku University Hospital in 1998 and 2008 and the Ethical Committee of the University of Turku in 2014 and 2018. Permission was obtained from school authorities individually for each study. Participation was voluntary for adolescents, and their anonymity was ensured throughout the study. According to the Finnish regulations, since the questionnaires were filled out anonymously, parental consent was not needed. Parents were, however, informed about the study, and they had the possibility to not allow their adolescent to participate.

### Measures

#### Demographic details

Information on age, gender, and family structure was collected. Family structure was divided into living with two biological parents and other types of families, including one biological parent, remarried parents, foster parents, and adoptive parents. Body mass index (BMI) was calculated based on self-reported weight (in kilograms) and height (in meters) squared. BMI was divided into three categories, separately for females and males. For females, the categories were < 16.1 (underweight), 16.1–24.0 (normal weight), and > 24.0 (overweight); and for males, they were < 15.9 (underweight), 15.9–22.4 (normal weight), and > 22.4 (overweight). The categories were based on a large study [28] in which the authors defined BMI cut-off points among a group of Finnish children for being thin, overweight or obese. If an adolescent’s measurement was right on the line (e.g., 16.1), they were classified into the higher BMI category. Girls were also asked whether menstruation had started or if menstruation had ended at some point.

#### Body dissatisfaction and dieting

Body dissatisfaction and dieting were measured with a ten-item Body Image and Eating Distress (BIEDS) scale [29]. This self-report instrument is used to assess attitudes and behaviors related to body shape and dieting. The items were derived from the DSM-IV criteria for anorexia nervosa and bulimia nervosa. The questionnaire included the following nine items: 1. I want to be thinner; 2. I exercise in order not to gain weight; 3. I have been dieting; 4. I am fearful of becoming fat; 5. I have lost weight during a short time because I have not been eating enough; 6. I am dissatisfied with my body; 7. I am scared if my weight increases even a little; 8. I can’t control my eating; 9. I have consumed large amounts of food at one time; 10. I practice self-induced vomiting after eating. Respondents were asked to indicate on a three-point scale their attitudes and behaviors over the last 6 months (0 = not true; 1 = somewhat true; 2 = certainly true). The sum of the items was used to generate an eating distress total score ranging from 0 to 18. Higher scores indicated more eating distress symptoms. We defined a cut-off point of total eating disturbance scale scores to define subjects with most severe symptoms. The cut-off point was if scores put a person in the 90th percentile in the 1998 data. The cut-off point was the same for males and females across all data collections.

In 2018, the instrument was validated at a university hospital in Finland, among 30 adolescents and young adults diagnosed with an eating disorder in an outpatient unit specified for eating disorders. The data consisted of adolescents and young adults who were treated in the Eating Disorder Clinic due to severe to moderate eating disorders. They had the diagnosis of anorexia nervosa, atypical anorexia nervosa or bulimia nervosa. They were asked to fill out the eating distress questionnaire as well as the Eating Disorder Examination Questionnaire (EDE-Q), which is valid and commonly used [30]. The two instruments’ sets of results were compared, and the psychometric evaluation showed that the instrument we used demonstrated good reliability and validity and had a good level of internal consistency. The correlations between our instrument and the EDE-Q varied between 0.72 and 0.81 for subscales and was 0.81 for total scales. The internal consistency (Cronbach’s alpha) was 0.81, thus well exceeding the recommended Cronbach’s alpha value of > 0.7. In the current study, the Cronbach’s alpha for females was 0.81 in 1998, 0.85 in 2008, 0.83 in 2014, and 0.83 in 2018 and for males 0.72, 0.74, 0.72, and 0.71, respectively.

### Statistical methods

The association between year and the total BIEDS score was examined using logistic regression. The dependent variable was the score as a binary variable, with a score less than 90% as a cut-off point (calculated from the 1998 sample) for the reference category. Changes in individual items were examined by multinomial logistic regression with the item as a dependent variable. The option “Not true” was used as a reference category so that the odds ratios (OR) and confidence intervals (CI) were estimated for “Somewhat true” vs. “Not true” and “Certainly true” vs. “Not true”. For the main analysis, we only included the years 1998 and 2018. The year of data collection was used as an explanatory variable and odds ratios with 95% confidence intervals were calculated for 1998 vs. 2018. For an adjusted analysis, school grade, family structure and city were included in the models as explanatory variables.

As a supplemental analysis, we repeated the models described above with data from all four years included. Odds ratios were calculated for 1998 vs. 2008, 2008 vs. 2014, 2014 vs. 2018, and 1998 vs. 2018. Because there were four odds ratios per outcome, we used Bonferroni correction to control the overall probability of false positive results. This meant that a p-value lower than 0.05/4 = 0.0125 was considered significant for one pairwise comparison, and odds ratios were calculated with a 98.75% confidence interval. Type 3 tests were performed to estimate the overall significance of the year as an explanatory variable. An overall p-value smaller than 0.05 was considered significant, meaning that the average outcome was not similar in all years.

The statistical analyses were carried out using SAS software, version 9.4 for Windows.

## Results

The participants of each study (1998, 2008, 2014, and 2018) were similar in age, school grade division, and gender, as presented in Table 1. A statistically significant time effect was found in height (*p* < 0.001), weight (*p* < 0.001), and BMI among females (*p* < 0.001) and males (*p* = 0.029). Height and weight increased steadily from 1998 to 2018. In 2018, the average height was 1.5 cm higher than in 1998, and the average weight was 3.5 kg higher. According to the significant time effect in BMI, there were fewer underweight and normal weight adolescents and more overweight adolescents in 2018 than in 1998.

**Table 1:** Percentages and mean values of background characteristics of participants

Characteristics	1998 (*N* = 1458)	2008 (*N* = 2044)	2014 (*N* = 1809)	2018 (*N* = 1349)
City n (%)				
Rovaniemi	807 (55.3)	1318 (64.5)	1075 (59.4)	770 (57.1)
Salo	651 (44.7)	726 (35.5)	734 (40.6)	579 (42.9)
Age (years)				
Mean (SD)	14.4 (1.1)	14.4 (1.1)	14.3 (1.1)	14.5 (1.1)
Min–max	13–17	13–17	13–17	13–17
School grade n (%)				
7th graders	727 (49.9)	1067 (52.2)	932 (51.5)	668 (49.5)
9th graders	731 (50.1)	977 (47.8)	877 (48.5)	681 (50.5)
Gender n (%)				
Females	725 (49.7)	1027 (50.2)	877 (48.5)	680 (50.4)
Males	733 (50.3)	1017 (49.8)	932 (51.5)	669 (49.6)
Family structure n (%)				
Family with two biological parents	1016 (70.4)	1345 (66.1)	1261 (70.5)	728 (70.5)
Other family	427 (29.6)	689 (33.9)	527 (29.5)	305 (29.5)
Height (cm)				
Min–max	130–198	139–210	140–197	142–201
Mean (SD)	165.8 (8.8)	166.7 (9.1)	167.0 (9.0)	167.3 (9.2)
Weight (kg)				
Min–max	30–116	30–120	30–120	34–125
Mean (SD)	55.4 (10.7)	57.7 (12.4)	57.9 (12.1)	58.9 (12.2)
Body mass n (%)				
Females				
< 16.1 (underweight)	30 (4.4)	45 (4.6)	34 (4.1)	17 (2.7)
16.1–24.0 (normal weight)	621 (90.9)	830 (84.0)	701 (84.4)	518 (83.7)
> 24.0 (overweight)	32 (4.7)	113 (11.4)	96 (11.6)	84 (13.6)
Males				
< 15.9 (underweight)	12 (1.7)	24 (2.5)	16 (1.8)	18 (2.9)
15.9–22.4 (normal weight)	540 (78.4)	691 (71.2)	645 (74.1)	446 (71.2)
> 22.4 (overweight)	137 (19.9)	256 (26.4)	209 (24.0)	162 (25.9)
Menstruation n (%)				
Has started	626 (88.3)	893 (87.8)	752 (87.7)	613 (90.1)
Has not started	76 (10.7)	115 (11.3)	96 (11.2)	60 (8.8)
Has ended	7 (1.0)	9 (0.9)	9 (1.1)	7 (1.0)

### Changes in body dissatisfaction and dieting

Tables 2 and 3 present the changes in total scores and in the frequency distribution of the self-reported body dissatisfaction and dieting by gender for years 1998 and 2018. Changes for each assessment year (1998, 2008, 2014, 2018) can be found in supplement 1a and 1b. The results for both females and males were adjusted for school grade, family structure, and city. After adjustment, the results remained rather unchanged.

**Table 2:** Comparison of self-reported body dissatisfaction and dieting among females between 1998 and 2018

	1998 *N* = 725	2018 *N* = 680	*p*-value^a^	OR (95%CI)^a^ 1998 vs. 2018
	%	%		
Total scores over cut-off^b^				
≥ = 90% Cut-off	21.2	14.0	**0.004**	**1.6 (1.15–2.10)**
Want to be thinner				
Not true	26.1	33.6		1
Somewhat true	37.4	43.0	0.384	1.1 (0.86–1.49)
Certainly true	36.6	23.4	** < 0.001**	**1.9 (1.42–2.58)**
I exercise a lot to avoid gaining weight				
Not true	23.6	40.6		1
Somewhat true	56.5	49.5	** < 0.001**	**2.0 (1.54–2.58)**
Certainly true	19.9	9.9	** < 0.001**	**3.0 (2.09–4.37)**
I have been on a diet				
Not true	59.7	76.7		1
Somewhat true	23.7	14.9	** < 0.001**	**1.9 (1.43–2.59)**
Certainly true	16.6	8.4	** < 0.001**	**2.3 (1.63–3.36)**
I am afraid of getting fat				
Not true	35.9	42.8		1
Somewhat true	34.4	34.2	0.096	1.3 (0.96–1.64)
Certainly true	29.7	23.0	**0.010**	**1.5 (1.09–1.94)**
I have lost weight considerably over a short period of time				
Not true	76.2	79.6		1
Somewhat true	16.6	13.4	0.263	1.2 (0.87–1.65)
Certainly true	7.2	6.9	0.991	1.0 (0.64–1.55)
I am not happy with my body			** < 0.001**	
Not true	21.0	31.8		1
Somewhat true	51.9	44.6	** < 0.001**	**1.6 (1.24–2.16)**
Certainly true	27.1	23.6	**0.002**	**1.6 (1.20–2.27)**
It terrifies me if I gain even a little weight				
Not true	53.2	63.6		1
Somewhat true	30.4	23.9	**0.009**	**1.4 (1.09–1.85)**
Certainly true	16.4	12.5	**0.038**	**1.4 (1.02–1.99)**
I am not always able to control my eating				
Not true	46.5	56.7		1
Somewhat true	38.3	32.6	**0.018**	**1.4 (1.05–1.73)**
Certainly true	15.2	10.7	**0.008**	**1.6 (1.13–2.31)**
I consume large amounts of food at one time				
Not true	59.5	72.4		1
Somewhat true	31.9	24.2	**0.001**	**1.5 (1.17–1.96)**
Certainly true	8.6	3.4	** < 0.001**	**2.9 (1.67–4.90)**
I have willfully vomited after eating				
Not true	93.4	92.3		1
Somewhat true	4.1	4.9	0.354	0.8 (0.45–1.33)
Certainly true	2.5	2.8	0.488	0.8 (0.39–1.56)

The significance level is < 0.05 (in bold)

^a^Adjusted for school grade, family structure and city

^b^The cut-off point was based on the highest 90th percentile scores of the total eating disturbance scale score in 1998. The same cut-off score was used for both females and males

**Table 3:** Comparison of self-reported body dissatisfaction and dieting among males between1998 and 2018

	1998 *N* = 733	2018 *N*=669	*p*-value^a^	OR (95%CI)^a^ 1998 vs. 2018
	%	%		
Total scores over cut-off ^b^				
≥ = 90% Cut-off	3.8	2.1	0.275	1.5 (0.74–2.85)
Want to be thinner				
Not true	63.1	63.7		1
Somewhat true	27.8	30.5	0.421	0.9 (0.70–1.16)
Certainly true	9.2	5.8	0.141	1.4 (0.89–2.18)
I exercise a lot to avoid gaining weight				
Not true	39.1	40.8		1
Somewhat true	41.3	43.5	0.624	0.9 (0.73–1.21)
Certainly true	19.6	15.7	0.242	1.2 (0.87–1.71)
I have been on a diet				
Not true	85.9	89.8		1
Somewhat true	11.1	8.2	0.123	1.4 (0.92–2.03)
Certainly true	3.0	2.0	0.261	1.5 (0.72–3.31)
I am afraid of getting fat				
Not true	76.3	76.2		1
Somewhat true	17.3	19.1	0.202	0.8 (0.62–1.11)
Certainly true	6.4	4.7	0.364	1.3 (0.76–2.10)
I have lost weight considerably over a short period of time				
Not true	85.6	88.6		1
Somewhat true	11.6	9.1	0.462	1.2 (0.79–1.68)
Certainly true	2.7	2.3	0.646	0.8 (0.41–1.73)
I am not happy with my body				
Not true	56.7	62.8		1
Somewhat true	32.6	28.4	0.167	1.2 (0.93–1.55)
Certainly true	10.7	8.7	0.367	1.2 (0.80–1.80)
It terrifies me if I gain even a little weight				
Not true	87.4	89.8		1
Somewhat true	10.4	8.4	0.440	1.2 (0.79–1.72)
Certainly true	2.2	1.8	0.833	0.9 (0.42–2.02)
I am not always able to control my eating				
Not true	66.7	69.9		1
Somewhat true	23.8	26.0	0.348	0.9 (0.67–1.15)
Certainly true	9.5	4.1	**0.012**	**1.9 (1.15–3.07)**
I consume large amounts of food at one time				
Not true	57.7	64.5		1
Somewhat true	32.0	31.2	0.420	1.1 (0.86–1.43)
Certainly true	10.3	4.3	**0.002**	**2.1 (1.30–3.41)**
I have willfully vomited after eating				
Not true	96.8	97.6		1
Somewhat true	1.4	1.8	0.115	0.5 (0.19–1.20)
Certainly true	1.8	0.6	0.300	1.8 (0.58–5.77)

The significance level is < 0.05 (in bold)

^a^Adjusted for school grade, family structure and city

^b^The cut-off point was based on the highest 90th percentile scores of the total eating disturbance scale score in 1998. The same cut-off score was used for both females and males

### Females

During the 20-year study period, from 1998 to 2018, there were significant decreases in body dissatisfaction and dieting among females. The number of females in the 90th percentile with the most severe symptoms decreased significantly over the 20 years (OR 1.6, 95% CI 1.15–2.10). We found a decrease in the number of females who were dissatisfied with their body (OR 1.6, 95% CI 1.24–2.16), females who wanted to be thinner (OR 1.9, 95% CI 1.42–2.58), and those who were terrified of even a small amount of weight gain (OR 1.5, 95% CI 1.09–1.94). The percentage of females who exercised a lot to avoid gaining weight also decreased (OR 3.0, 95% CI 2.09–4.37) as did that of those who had been on diet (OR 2.3, 95% CI 1.63–3.36) and those who had problems controlling their eating (OR 1.6, 95% CI 1.13–2.31). Finally, fewer females reported consuming large amounts of food at one time (OR 2.9, 95% CI 1.67–4.90). A more detailed comparison of the self-reported body dissatisfaction and dieting among females between assessment years is presented in Table 2.

### Males

When comparing the data from 1998 and 2018 for males, values for almost every item remained fairly stable (Table 3).

**Fig. 2:**
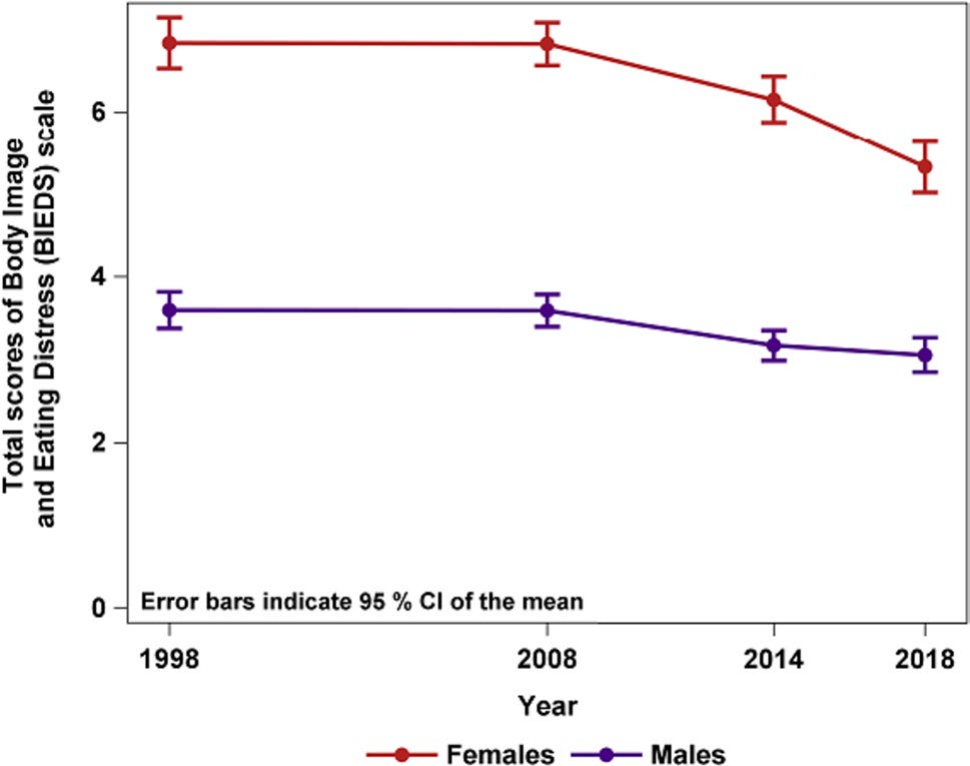
Plot of gender and year for means of total scores (sum of items 1–9) of self-reported body dissatisfaction and dieting

As shown in Fig. 2, between 1998 and 2018, the total scores of BIEDS decreased among females but not among males. In addition, the number of females who had not been on diet increased 17.0 percentage points and females not being dissatisfied with their body increased 10.8 percentage points between the first and last assessment point.

**Fig. 3:**
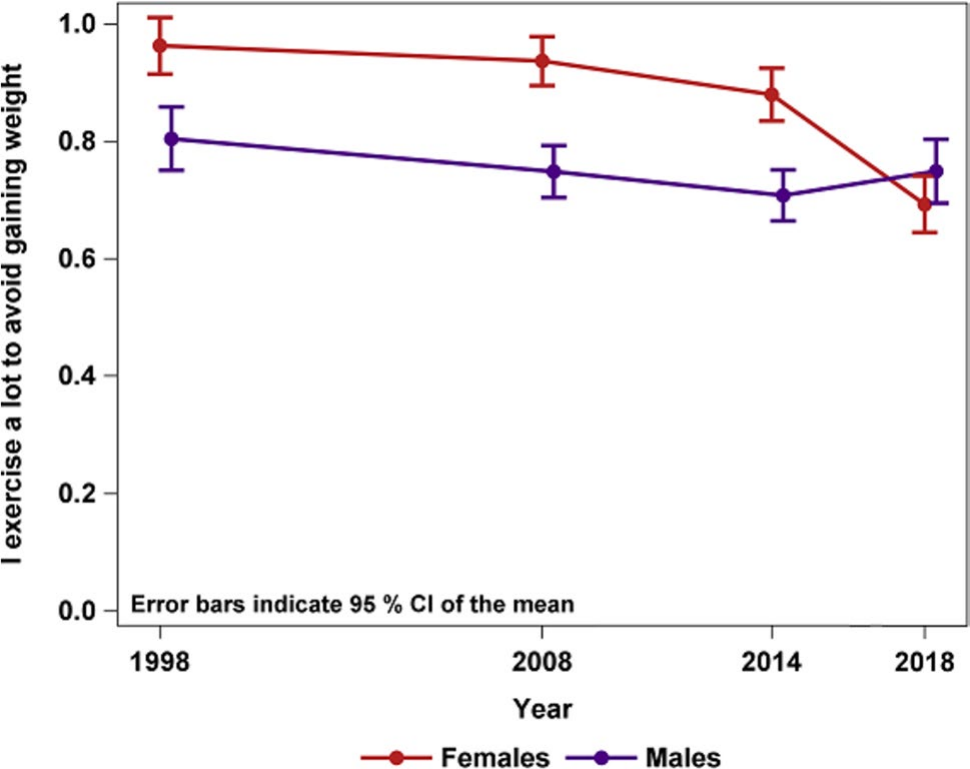
Interaction plot of gender and year for exercising to control weight gain

According to multinomial logistic regression, there was an interaction between year and gender regarding exercise to control weight (interaction sex × year *p* < 0.001). The interaction was tested for all years. As shown in Fig. 3, there was a continuous decrease in the proportion of females exercising to avoid gaining weight (i.e., those respondents who responded to this item anything other than ”not true”) with every assessment: 76.4% in 1998, 73.6% in 2008, 70.6% in 2014, and 59.4% in 2018. The corresponding proportion of males, however, remained rather stable over time: 60.9% in 1998, 58.4% in 2008, 58.4% in 2014, and 59.2% in 2018 (Table 2 and supplement 1). Females reported exercising to avoid weight gain more than males until 2018, when both genders reported similar levels of exercising.

## Discussion

Using data from a repeated cross-sectional school-based survey conducted in Finnish secondary schools, we found a stable and decreasing trend in self-reported body dissatisfaction and dieting between 1998 and 2018. The main finding was that body dissatisfaction among adolescent females improved over the 20-year study period. We did not observe this type of trend among males as their situation was rather stable. This seems to contradict concerns that the growing use of social media is negatively impacting body image [31]. Our first main finding contradicts our hypothesis that body dissatisfaction would be more stable than improved. One potential reason is that this study is based on more current data than the previous Finnish study [22], which was a central base for our hypothesis. The second main finding was that self-perceived dieting remained relatively stable.

There was a significant decrease in the prevalence of females reporting dissatisfaction with their body during the 20-year study period. Among males, the rates did not fluctuate much during the same period. Males also reported less body dissatisfaction than the females at every assessment point. Our results align with some previous studies and are based on more current data. Large studies by Asgeirdottir et al. [20] and Ingolfssdottir et al. [21] with nationally representative samples in Iceland reported improved body image among adolescents with more pronounced positive changes for females than males. A more stable trend was found by Hadjigeorgiou et al. [17] in Cyprus, reporting no changes in the frequency of pathological scoring for thinness or body dissatisfaction.

The results of the current study are interesting since it has been reported that traditional media and social media has mostly negatively influenced body image among adolescent females [32–34]. The reported trend toward lower body dissatisfaction in our study despite the extensive and increasing use of social media [35, 36] may give another perspective to this. For example, a review [26] indicates that social media usage may lead to body image and eating difficulties. Our findings may further suggest that the association between social media use and body image can also be different [37] as the content can also promote body positivity. In fact, daily exposure to body-positive content on social media is known to be associated with increased body satisfaction [38]. However, social media usage was not asked about in our study, so these assumptions are based on general rates of social media usage. It is also possible that the trend of decreasing body dissatisfaction is due to other factors, such as the social movement of body positivity and attempts to restrict unrealistic images of bodies in the fashion business [39]. Moreover, the body-positive movement is being popularized through social media platforms like Instagram [37], and the movement seems to be continuing to grow in popularity and reach [40]. Thus, body positivity on social media can serve as a platform for promoting body positivity on a population level [40].

Changes in exercise to avoid weight gain were found among females. Exercising to avoid gaining weight decreased, and the proportion of females who did not exercise to avoid gaining weight increased from 24 to 41%. Although this result only tells us about exercising with the motivation of avoiding weight gain, the situation of adolescents’ physical activity in general is worrisome. Global trends show that the majority of adolescents do not meet current physical activity guidelines [41]. Moreover, a large population-based survey among Norwegian adolescents reported that a low proportion of adolescents are sufficiently physically active, but that their physical activity levels have been fairly stable between 2005 and 2018 [42]. This may indicate that, although physical activity itself might be stable, the reasons for exercising have changed over the years. Nevertheless, there is a need to develop and implement ways to increase physical activity among adolescents [41], but with healthy motivations.

The results are indeed positive, especially considering the wellbeing of females. However, despite this and the possibility of an even narrower gap in body dissatisfaction between genders in the future, as much as almost 70% of the females in our study sample were still dissatisfied with their body (i.e., those respondents who responded to the item ”I am not happy with my body” anything other than ”not true” in 2018). Also, when comparing dieting and body dissatisfaction between males and females, females have many more problems than males, although the gap seems to be narrowing. Moreover, despite the positive changes presented here, it is worth noticing that every three out of four girls at least sometimes wished to be thinner, although only 14% of our study sample were overweight.

Overall, the adolescents’ reports of eating distress during the 20-year study period remained fairly stable. This finding is in line with another Finnish study by Litmanen et al. [22], which reported stable trends from 2002 to 2013 among 15-year-old adolescents. They found no change in the proportion of adolescents with an eating disorder or eating disorder symptoms. One of our positive findings was the decrease from 2014 to 2018 in the proportion of females on a diet. Before 2014, the stayed quite stable. In three samples of Norwegian 16–17-year-old adolescents, von Soest et al. [23] found increases in dieting from 1992 to 2010. Likewise, a Swedish study found increasing numbers of dieting among 11–15-year-old females and males from 1994 to 2014 [24]. Our study saw decreases in the number of dieting females, especially between the last two measurement points, from 2014 to 2018. It is possible that a decrease in dieting is a new trend and was not yet seen in the aforementioned studies, and that similar trends might be found in future studies.

### Limitations

The following limitations should be considered when interpreting the results. The data were based on the self-reporting of adolescents and the information would have been more extensive if combined with parents’ ratings. However, collecting self-reports on an anonymous basis in sealed envelopes was the best available way to gather this information. No diagnostic interviews were used in the assessment. However, the study also concentrated on adolescents’ assessments of their body dissatisfaction and dieting before their symptoms exceeded diagnostic levels. Therefore, self-reports measuring several aspects of eating and body dissatisfaction were in focus here. Limitations also include the focus of the instrument that was used. The instrument targets body dissatisfaction and dieting with a focus on pathology and risk of disordered behavior, rather than on health promotion, which limits the interpretation of the participants’ wellbeing. In addition, some of the formulations of the items may have affected the results. For example, “Exercise to reduce body weight” or “Exercise to alter body shape” would have likely produced different results than “Exercise to avoid weight gain”. The response rates remained at almost the same level each year, with approximately 10–15% of the possible respondents not taking part in the study. Most of the non-respondents were absent from school on the day of the survey, even though the teachers were instructed to ask them to fill in the questionnaire later. Since school absenteeism is associated with increased mental health problems [43], it is possible that we have missed adolescents with more or more severe problems. However, since the study was conducted using similar methods each time, it is likely that absenteeism is present similarly in each assessment point. Therefore, this issue does not make the assessment of change non-reliable.

There are also several strengths in our study. The data were collected from two medium-sized cities, one in the north and one in the south of Finland, that comprised both urban and rural areas and reflected the composition of typical Finnish cities. The demographical characteristics of the participants, such as sex distribution, ethnic background and family composition of the communities, were comparable to the national statistics for that age group in Finland. Our results can therefore be generalized to Finnish society, but not to other countries. Hence, it is important that further studies be conducted in other countries and social contexts. No personal details, such as names, were sought so that the respondents would feel that they could answer as freely as possible; this increases the reliability of the findings. In addition, the instrument was validated, and the psychometric evaluation showed that the instrument demonstrated good reliability and validity, and a good level of internal consistency.

## Conclusions

Our findings are encouraging and suggest that body dissatisfaction and dieting have decreased among Finnish adolescents since 1998. This may be indicative of the growing trend of body positivity that promotes acceptance and appreciation of all body sizes and appearances. However, despite the positive changes, especially among females, dieting and body dissatisfaction are still common and more prevalent among females.

### Supplementary Information

Below is the link to the electronic supplementary material.

Supplementary file1 (DOCX 45 KB)

## Data Availability

The data that support the findings of this study are available from the last author, [AS], upon reasonable request.
