# Supplementary material for: Body dissatisfaction and dieting among Finnish adolescents: a 20-year population-based time-trend study
Source: Eur Child Adolesc Psychiatry. 2024 Jan 2;33(8):2605–14. doi: 10.1007/s00787-023-02327-0 (PMC11272674; doi:10.1007/s00787-023-02327-0)
Supplement: Supplementary file 1 — Supplementary file1 (DOCX 45 KB) [file 787_2023_2327_MOESM1_ESM.docx]

**Supplement 1a.** Comparison of self-reported body dissatisfaction and dieting between assessment years. Females

|  | **1998**  **N=725** | **2008**  **N=1027** | **2014**  **N=932** | **2018**  **N=669** | **Adjusted**  **overall**  **year**  ***p* value^a^** | **Adjusted p value of pairwise year comparisons**  **OR (98.75%CI)^a^** | | | |
| --- | --- | --- | --- | --- | --- | --- | --- | --- | --- |
|  | **%** | **%** | **%** | **%** |  | **1998 vs. 2008** | **2008 vs. 2014** | **2014 vs. 2018** | **1998 vs. 2018** |
| **Total scores** **over cut-off ^b^** | | | | | **0.011** |  |  |  |  |
| ≥=90% Cut-off | 21.2 | 19.3 | 16.8 | 14.0 |  | 1.1 (0.85-1.56) | 1.2 (0.86-1.58) | 1.2 (0.81-1.74) | **1.6 (1.08-2.33)** |
| **Want to be thinner** | | | | | **<0.001** |  |  |  |  |
| Not true | 26.1 | 23.4 | 27.0 | 33.6 |  |  |  |  |  |
| Somewhat true | 37.4 | 38.2 | 40.6 | 43.0 |  | 0.9 (0.65-1.21) | 1.1 (0.80-1.44) | 1.2 (0.86-1.68) | 1.1 (0.81-1.62) |
| Certainly true | 36.6 | 38.4 | 32.4 | 23.4 |  | 0.9 (0.63-1.19) | 1.3 (0.99-1.81) | **1.7 (1.16-2.41)** | **1.9 (1.33-2.84)** |
| **I exercise a lot to avoid gaining weight** | | | | | **<0.001** |  |  |  |  |
| Not true | 23.6 | 26.4 | 29.4 | 40.6 |  |  |  |  |  |
| Somewhat true | 56.5 | 53.5 | 53.2 | 49.5 |  | 1.2 (0.88-1.58) | 1.1 (0.86-1.47) | **1.5 (1.10-2.03)** | **2.0 (1.43-2.74)** |
| Certainly true | 19.9 | 20.1 | 17.4 | 9.9 |  | 1.1 (0.76-1.58) | 1.3 (0.91-1.83) | **2.1 (1.36-3.37)** | **3.0 (1.91-4.87)** |
| **I have been on a diet** | | | | | **<0.001** |  |  |  |  |
| Not true | 59.7 | 60.8 | 67.5 | 76.7 |  |  |  |  |  |
| Somewhat true | 23.7 | 24.1 | 19.6 | 14.9 |  | 1.0 (0.76-1.37) | **1.4 (1.02-1.82)** | 1.4 (0.98-2.08) | **2.0 (1.35-2.88)** |
| Certainly true | 16.6 | 15.1 | 12.9 | 8.4 |  | 1.1 (0.80-1.59) | 1.3 (0.91-1.81) | **1.6 (1.01-2.51)** | **2.3 (1.46-3.63)** |
| **I am afraid of getting fat** | | | | | **<0.001** |  |  |  |  |
| Not true | 35.9 | 26.0 | 31.9 | 42.8 |  |  |  |  |  |
| Somewhat true | 34.4 | 36.7 | 37.8 | 34.2 |  | **0.7 (0.51-0.92)** | 1.2 (0.88-1.56) | **1.5 (1.11-2.13)** | 1.2 (0.88-1.72) |
| Certainly true | 29.7 | 37.3 | 30.3 | 23.0 |  | **0.6 (0.43-0.79)** | **1.5 (1.12-2.01)** | **1.7 (1.18-2.39)** | **1.5 (1.02-2.09)** |
| **I have lost weight considerably over  a short period of time** | | | | | 0.131 |  |  |  |  |
| Not true | 76.2 | 78.6 | 81.7 | 79.6 |  |  |  |  |  |
| Somewhat true | 16.6 | 14.0 | 13.1 | 13.4 |  | 1.3 (0.89-1.76) | 1.1 (0.78-1.55) | 0.9 (0.59-1.32) | 1.2 (0.81-1.82) |
| Certainly true | 7.2 | 7.4 | 5.1 | 6.9 |  | 1.0 (0.64-1.64) | 1.5 (0.89-2.37) | 0.7 (0.37-1.16) | 1.0 (0.56-1.70) |
| **I am not happy with my body** | | | | | **<0.001** |  |  |  |  |
| Not true | 21.0 | 23.0 | 28.5 | 31.8 |  |  |  |  |  |
| Somewhat true | 51.9 | 47.0 | 44.3 | 44.6 |  | 1.2 (0.89-1.66) | 1.3 (0.97-1.73) | 1.0 (0.75-1.45) | **1.6 (1.15-2.33)** |
| Certainly true | 27.1 | 30.0 | 27.2 | 23.6 |  | 1.0 (0.71-1.43) | 1.3 (0.98-1.84) | 1.2 (0.83-1.77) | **1.6 (1.10-2.47)** |
| **It terrifies me if I gain even a little weight** | | | | | **0.003** |  |  |  |  |
| Not true | 53.2 | 50.6 | 56.2 | 63.6 |  |  |  |  |  |
| Somewhat true | 30.4 | 31.7 | 26.6 | 23.9 |  | 0.9 (0.70-1.22) | **1.3 (1.01-1.72)** | 1.2 (0.85-1.63) | **1.4 (1.02-2.00)** |
| Certainly true | 16.4 | 17.7 | 17.2 | 12.5 |  | 0.9 (0.64-1.27) | 1.1 (0.82-1.55) | 1.4 (0.95-2.15) | 1.5 (0.95-2.23) |
| **I am not always able to control my eating** | | | | | **<0.001** |  |  |  |  |
| Not true | 46.5 | 52.6 | 57.5 | 56.7 |  |  |  |  |  |
| Somewhat true | 38.3 | 33.2 | 32.5 | 32.6 |  | **1.3 (1.01-1.72)** | 1.1 (0.86-1.43) | 0.9 (0.68-1.25) | 1.3 (0.98-1.84) |
| Certainly true | 15.2 | 14.2 | 10.0 | 10.7 |  | 1.2 (0.85-1.75) | **1.5 (1.05-2.23)** | 0.9 (0.55-1.37) | **1.6 (1.03-2.54)** |
| **I consume large amounts of food at one time** | | | | | **<0.001** |  |  |  |  |
| Not true | 59.5 | 71.6 | 70.1 | 72.4 |  |  |  |  |  |
| Somewhat true | 31.9 | 22.6 | 25.8 | 24.2 |  | **1.7 (1.29-2.26)** | 0.8 (0.65-1.12) | 1.0 (0.76-1.43) | **1.5 (1.09-2.09)** |
| Certainly true | 8.6 | 5.9 | 4.1 | 3.4 |  | **1.8 (1.10-2.87)** | 1.4 (0.79-2.36) | 1.2 (0.57-2.46) | **2.9 (1.45-5.66)** |
| **I have willfully vomited after eating** | | | | | 0.923 |  |  |  |  |
| Not true | 93.4 | 92.4 | 92.5 | 92.3 |  |  |  |  |  |
| Somewhat true | 4.1 | 4.7 | 4.2 | 4.9 |  | 0.9 (0.51-1.68) | 1.0 (0.59-1.83) | 0.8 (0.43-1.58) | 0.8 (0.40-1.56) |
| Certainly true | 2.5 | 2.9 | 3.3 | 2.8 |  | 0.9 (0.41-1.87) | 0.8 (0.43-1.63) | 1.1 (0.48-2.35) | 0.8 (0.33-1.88) |

**^a^** Adjusted for school grade, family structure and city.

**^b^** The cut-off point was based on the highest 90^th^ percentile scores of the total eating disturbance scale score in 1998. The same cut-off score was used for both females and males.

**Supplement 1b.** Comparison of self-reported body dissatisfaction and dieting between assessment years. Males

|  | **1998**  **N=733** | **2008**  **N=1017** | **2014**  **N=932** | **2018**  **N=669** | **Adjusted**  **overall**  **year**  ***p* value^a^** | **Adjusted p value of pairwise year comparisons**  **OR (98.75%CI)^a^** | | | |
| --- | --- | --- | --- | --- | --- | --- | --- | --- | --- |
|  | **%** | **%** | **%** | **%** |  | **1998 vs. 2008** | **2008 vs. 2014** | **2014 vs. 2018** | **1998 vs. 2018** |
| **Total scores** **over cut-off ^b^** | | | | | 0.102 |  |  |  |  |
| ≥=90% Cut-off | 3.8 | 4.6 | 2.7 | 2.1 |  | 0.8 (0.45-1.55) | 1.7 (0.91-3.22) | 1.0 (0.44-2.48) | 1.5 (0.64-3.51) |
| **Want to be thinner** | | | | | **0.013** |  |  |  |  |
| Not true | 63.1 | 58.0 | 61.8 | 63.7 |  |  |  |  |  |
| Somewhat true | 27.8 | 30.2 | 30.0 | 30.5 |  | 0.8 (0.63-1.10) | 1.1 (0.86-1.43) | 1.0 (0.71-1.32) | 0.9 (0.64-1.23) |
| Certainly true | 9.2 | 11.9 | 8.1 | 5.8 |  | 0.7 (0.47-1.07) | **1.6 (1.06-2.36)** | 1.2 (0.71-2.17) | 1.4 (0.79-2.45) |
| **I exercise a lot to avoid gaining weight** | | | | | **0.007** |  |  |  |  |
| Not true | 39.1 | 41.7 | 41.6 | 40.8 |  |  |  |  |  |
| Somewhat true | 41.3 | 41.9 | 46.0 | 43.5 |  | 1.1 (0.81-1.40) | 0.9 (0.71-1.18) | 1.0 (0.71-1.31) | 0.9 (0.68-1.30) |
| Certainly true | 19.6 | 16.5 | 12.4 | 15.7 |  | 1.3 (0.92-1.86) | 1.3 (0.92-1.89) | 0.7 (0.47-1.11) | 1.2 (0.81-1.91) |
| **I have been on a diet** | | | | | 0.473 |  |  |  |  |
| Not true | 85.9 | 88.7 | 88.4 | 89.8 |  |  |  |  |  |
| Somewhat true | 11.1 | 8.7 | 9.0 | 8.2 |  | 1.4 (0.90-2.04) | 1.0 (0.64-1.43) | 1.0 (0.63-1.70) | 1.3 (0.81-2.21) |
| Certainly true | 3.0 | 2.7 | 2.6 | 2.0 |  | 1.1 (0.55-2.39) | 1.0 (0.50-2.09) | 1.4 (0.53-3.57) | 1.6 (0.61-4.24) |
| **I am afraid of getting fat** | | | | | **<0.001** |  |  |  |  |
| Not true | 76.3 | 66.9 | 73.2 | 76.2 |  |  |  |  |  |
| Somewhat true | 17.3 | 23.0 | 21.4 | 19.1 |  | **0.7 (0.49-0.91)** | 1.2 (0.90-1.57) | 1.0 (0.73-1.47) | 0.8 (0.57-1.20) |
| Certainly true | 6.4 | 10.1 | 5.3 | 4.7 |  | **0.6 (0.36-0.92)** | **2.1 (1.30-3.28)** | 1.1 (0.57-2.05) | 1.3 (0.67-2.43) |
| **I have lost weight considerably over**  **a short period of time** | | | | | 0.681 |  |  |  |  |
| Not true | 85.6 | 86.4 | 88.8 | 88.6 |  |  |  |  |  |
| Somewhat true | 11.6 | 10.4 | 8.8 | 9.1 |  | 1.1 (0.72-1.58) | 1.2 (0.81-1.78) | 0.9 (0.56-1.47) | 1.2 (0.72-1.88) |
| Certainly true | 2.7 | 3.3 | 2.4 | 2.3 |  | 0.8 (0.38-1.68) | 1.4 (0.68-2.83) | 0.8 (0.32-1.86) | 0.9 (0.34-2.12) |
| **I am not happy with my body** | | | | | **0.002** |  |  |  |  |
| Not true | 56.7 | 52.8 | 58.7 | 62.8 |  |  |  |  |  |
| Somewhat true | 32.6 | 32.6 | 31.6 | 28.4 |  | 0.9 (0.72-1.24) | 1.2 (0.89-1.49) | 1.1 (0.79-1.48) | 1.2 (0.86-1.64) |
| Certainly true | 10.7 | 14.6 | 9.7 | 8.7 |  | **0.7 (0.45-0.98)** | **1.7 (1.19-2.51)** | 1.1 (0.64-1.74) | 1.2 (0.73-2.02) |
| **It terrifies me if I gain even a little weight** | | | | | 0.252 |  |  |  |  |
| Not true | 87.4 | 87.1 | 89.6 | 89.8 |  |  |  |  |  |
| Somewhat true | 10.4 | 9.3 | 8.5 | 8.4 |  | 1.1 (0.72-1.65) | 1.2 (0.77-1.74) | 0.9 (0.56-1.49) | 1.2 (0.71-1.90) |
| Certainly true | 2.2 | 3.7 | 2.0 | 1.8 |  | 0.6 (0.29-1.33) | 1.9 (0.91-3.90) | 0.9 (0.32-2.26) | 1.0 (0.37-2.69) |
| **I am not always able to control my eating** | | | | | **0.002** |  |  |  |  |
| Not true | 66.7 | 70.8 | 70.4 | 69.9 |  |  |  |  |  |
| Somewhat true | 23.8 | 23.3 | 25.5 | 26.0 |  | 1.1 (0.80-1.43) | 0.9 (0.71-1.22) | 0.9 (0.65-1.24) | 0.9 (0.63-1.25) |
| Certainly true | 9.5 | 5.8 | 4.1 | 4.1 |  | **1.7 (1.07-2.80)** | 1.3 (0.78-2.32) | 0.8 (0.41-1.60) | **1.9 (1.01-3.52)** |
| **I consume large amounts of food at one time** | | | | | **<0.001** |  |  |  |  |
| Not true | 57.7 | 66.0 | 64.6 | 64.5 |  |  |  |  |  |
| Somewhat true | 32.0 | 27.6 | 30.5 | 31.2 |  | **1.3 (1.01-1.75)** | 0.9 (0.68-1.13) | 1.0 (0.70-1.29) | 1.1 (0.81-1.53) |
| Certainly true | 10.3 | 6.3 | 31.2 | 4.3 |  | **1.8 (1.15-2.90)** | 1.3 (0.77-2.15) | 0.9 (0.47-1.76) | **2.1 (1.16-3.93)** |
| **I have willfully vomited after eating** | | | | | 0.080 |  |  |  |  |
| Not true | 96.8 | 98.0 | 97.5 | 97.6 |  |  |  |  |  |
| Somewhat true | 1.4 | 1.1 | 2.0 | 1.8 |  | 1.1 (0.34-3.56) | 0.5 (0.20-1.37) | 0.8 (0.31-2.17) | 0.5 (0.15-1.52) |
| Certainly true | 1.8 | 0.9 | 0.5 | 0.6 |  | 2.1 (0.68-6.29) | 1.5 (0.38-6.27) | 0.6 (0.11-3.32) | 2.0 (0.46-8.37) |

**^a^** Adjusted for school grade, family structure and city.

**^b^** The cut-off point was based on the highest 90^th^ percentile scores of the total eating disturbance scale score in 1998. The same cut-off score was used for both females and males.
